# Foraging trip duration of honeybee increases during a poor air quality episode and the increase persists thereafter

**DOI:** 10.1002/ece3.7145

**Published:** 2021-01-23

**Authors:** Yoori Cho, Sujong Jeong, Dowon Lee, Sang‐Woo Kim, Rokjin J. Park, Luke Gibson, Chunmiao Zheng, Chan‐Ryul Park

**Affiliations:** ^1^ Department of Environmental Planning Graduate School of Environmental Studies Seoul National University Seoul Korea; ^2^ School of Earth and Environmental Sciences Seoul National University Seoul Korea; ^3^ School of Environmental Science and Engineering Southern University of Science and Technology Shenzhen China; ^4^ Urban Forests Research Center National Institute of Forest Services Seoul Korea

**Keywords:** air pollution, air quality, airborne particulate matter, *Apis mellifera*, Depolarization Ratio, foraging, PM2.5, pollination, sky polarization

## Abstract

Increased concentration of airborne particulate matter (PM) in the atmosphere alters the degree of polarization of skylight which is used by honeybees for navigation during their foraging trips. However, little has empirically shown whether poor air quality indeed affects foraging performance (foraging trip duration) of honeybee. Here, we show apparent increases in the average duration of honeybee foraging during and after a heavy air pollution event compared with that of the pre‐event period. The average foraging duration of honeybees during the event increased by 32 min compared with the pre‐event conditions, indicating that 71% more time was spent on foraging. Moreover, the average foraging duration measured after the event did not recover to its pre‐event level. We further investigated whether an optical property (Depolarization Ratio, DR) of dominant PM in the atmosphere and level of air pollution (fine PM mass concentration) affect foraging trip duration. The result demonstrates the DR and fine PM mass concentration have significant effects on honeybee foraging trip duration. Foraging trip duration increases with decreasing DR while it increases with increasing fine PM mass concentration. In addition, the effects of fine PM mass concentration are synergistic with overcast skies. Our study implies that poor air quality could pose a new threat to bee foraging.

## Graphical abstract

The average foraging duration of honeybees during the event increased by 32 min compared with the pre‐event conditions, indicating that 71% more time is spent on foraging.

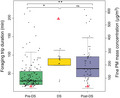

## 1 INTRODUCTION

Human food security is ensured by increasing global crop production, which is highly dependent on animal pollination (Garibaldi et al., 2009; Potts et al., 2016). The production of more than 70% of globally important commercial crop types is enhanced by animal pollinators (Klein et al., 2007). More fundamentally, the reproduction of approximately 87.5% of global flowering plant species relies on insect pollination (Ollerton et al., 2011). In addition to this important plant‐pollinator relationship, countless ecosystem services derive from pollination (Christmann, 2019; Kremen et al., 2007; Lundin et al., 2012). For example, pollinator‐dependent plant species such as *Rosa canica* and *Cornus mas*, are often used for erosion control (Comino & Marengo, 2010). Rapidly growing tulip poplar (*Liriodendron tulipifera* L.) is widely used for timber production and has a high capacity of carbon storage (Han et al., 2016).

Among many insect pollinators, bees are considered the key agents and contributors to pollination service (Powney et al., 2019). The population of bees, however, is declining globally due to various biotic and abiotic factors (Cappa et al., 2019; Klein et al., 2017; Ricketts et al., 2008). Anthropogenic stressors, especially that bees are vulnerable to have been identified and investigated (Klein et al., 2017; Potts et al., 2010). Some of the stressors include a lack of food resources (Goulson et al., 2015), pathogens, parasites (Goulson & Hughes, 2015), and pesticide use (Lundin et al., 2015; Woodcock et al., 2017).

While a substantial amount of research on bee population decline is focused on pesticide use, very few studies have assessed the impacts of air quality on pollinator activity. It is only recently that studies on relations between the olfactory learning of honeybees and air pollution have made progress. However, the potential effects of poor air quality on “honeybee vision” important for stable foraging (Srinivasan, 2011) are yet to be investigated.

Though invisible to the human eye, the polarization pattern of the e‐vector of skylight is utilized as a reliable compass for several pollinating insects (Foster et al., 2014), including the honeybee (*Apis mellifera*). Honeybees orient themselves and navigate between food sources and their hive by detecting polarized light patterns (Evangelista et al., 2014; Kraft et al., 2011; Rossel & Wehner, 1982). When the sun is occluded by cloud, they are still able to navigate by making use of polarization pattern around the sun. This celestial compass is considered to be the primary mechanism for orientation information (Dovey et al., 2013).

Polarized skylight information, therefore, should be provided sufficiently for honeybee to use (Rossel & Wehner, 1984). In natural scenes, the degree (or intensity) of polarization (DoP) values range from 0% to 50% in general (Foster et al., 2017). The threshold value for the degree of (linear) polarization that the honeybee needs for navigation can be as low as 10% (Brines & Gould, 1982). In an earlier observational study (Von Frisch, 1967), light polarization more than 15% assured well‐oriented waggle dances of bees. While the DoP under an aerosol‐free clean sky is generally strong, it decreases when nongaseous particles in the atmosphere additionally scatter the skylight (Labhart, 1996). The DoP can be extremely low, falling to zero when the atmosphere is heavily polluted by, for example, a massive dust storm (Zhao et al., 2018).

Thus, poor air quality could potentially be a major constraint for bee foraging and ultimately their contribution to pollination. Especially, climate change‐induced air pollution over South Asia and Southeast Asia is projected to deteriorate air quality in future (Kumar et al., 2018; Nguyen et al., 2019). Moreover, agricultural production in those continents and their countries is highly dependent on pollination service (Potts et al., 2016).

In this study, we aim to assess the impacts of air quality on honeybee foraging performance in specific ways that could also influence pollination efficiency. With a lack of direct measurement of real‐time DoP, changes in both optical property of dominant particles in the atmosphere and fine PM mass concentration are used as proxy for DoP alterations. Given that mean foraging trip durations were shown consistent in several studies, shorter than an hour at most (Colin et al., 2019; Higginson et al., 2011; Okubo et al., 2020; Perry et al., 2015), we hypothesize that honeybee foraging trip duration increases as a value of optical property of the atmosphere (Depolarization Ratio, DR) increase. We also hypothesize that an increase in foraging duration is strongly associated with ambient fine PM mass concentration.

## 2 MATERIAL AND METHODS

### 2.1 Experimental setting

To conduct this research with a field‐realistic approach, a domesticated honeybee colony in Beijing, China was monitored from April 27 to May 7, 2017. We monitored this honeybee colony and its foraging activities during an Asian dust event and quantified colony activity deviation from pre‐ and postdust storm event periods. 400 forager bees carried a Radio Frequency Identification (RFID) tag affixed to their thorax during the study time span.

A massive Asian dust storm originated from Central and East Asian deserts in early May 2017, blanketed various East Asian countries. Hourly PM10 and fine PM (with diameters < 2.5 µm) mass concentrations exceeded 1,000 µg/m^3^ and 250 µg/m^3^ in Beijing, respectively, and the air quality was observed to be at its worst on May 4 (Zhang et al., 2018). The aerosol optical depth (AOD) exceeded 2.1 (measured at 500 nm), and the particle DR from soil dust particles exceeded 0.3 (as average of up to 0.72 km altitude). There was a strong correlation between PM10 and fine PM mass concentrations throughout the study period (*r* = 0.97, *p* < .0001) and on the day of the dust episode (Zhang et al., 2018).

### 2.2 Study site

The experiment was conducted in an apiary on a hill bordering the Beijing Botanical Garden (BBG), Xiangshan park, Haidian District, Beijing, China (40°0′35″N, 116°12′2″E). The apiary is surrounded by mountains and the BBG within 1 km radius. It is located approximately 20 km north‐west of the city center. The BBG is one of the largest ex situ botanical garden in Beijing. It has approximately 6,000 plant species in a 56 ha area. Throughout the study period, pollinator‐dependent flowering species such as *Malus spectabilis*, *Rosa chinensis*, and *Iris sanguinea* were in full‐bloom. The apiary is managed by the Institute of Apicultural Research, Chinese Academy of Agricultural Sciences. A colony of approximately 20,000 honeybees including a single queen in a standard Langstroth hive was acquired from the apiary.

### 2.3 RFID monitoring

To monitor foraging trip durations of worker bees, bees were tagged with radio frequency identification (RFID) transponders (mic3^®^ ‐TAG 16k, microsensys GmbH, Erfurt, Germany). The square‐shaped transponder has dimensions of 2 × 1.7 × 0.5 mm, and weigh under 5 mg. RFID tags were glued to the thorax of 400 worker bees of mixed ages. Individual worker bees were given a unique identification number (UID) that was stored in each RFID tag. When a tagged bee passed a reader (MAJA reader module 4.1) installed at the entrance of the hive, the UID of the bee was read and the corresponding timestamp was stored in a host computer. Using the two recorded time points for the outbound and inbound trip, the foraging trip duration as time difference between the two trips could be calculated. Trip durations between 10 min and 250 min were selected and analyzed, as trips out of this range are considered either orientation flights or incomplete (Biesmeijer & Seeley, 2005; Degen et al., 2015). During the study period, 74,104 observation (outbound‐inbound trip in total) data were recorded in total. However, due to overlapping timestamps incurred by traffics at the hive entrance, durations for only 181 “identifiable” foraging trips in pairs (i.e., 362 timestamps) were used for statistical analysis. The monitoring of foraging activities for a colony with the RFID system occurred from April 27 to May 7, 2017.

### 2.4 Optical property data

Though the DoP can be measured by remote sensing sensors or polarimetric photography (Chen et al., 2020), it is difficult to obtain real‐time DoP data in compliance with our monitoring. The DR at 532 nm of Beijing (39.977°N, 116.381°E) measured by Mie‐scattering lidar was adopted to examine the effects of ambient aerosols on light polarization patterns. Values measured from 0.06 km to 0.72 km altitude, which were the lowest and highest altitudes in common with the DR measured throughout the observation days, were averaged and used. The DR is a reliable surrogate to suggest particulate matter irregularity (Pan et al., 2017). The irregular morphology of particles is important in terms of degree of light polarization. A larger DR (e.g., higher than 0.1) indicates that nonspherical particles are dominant in the atmosphere (Kim et al., 2010; Shimizu et al., 2016). Since honeybee estimates distances and directions between their nest and floral resources during their outbound trip (Evangelista et al., 2014), we used values of the optical property variable (DR) and fine PM concentration measured at the approximate times when bees started to forage. However, as DR and fine PM mass concentration are measured quarter‐hourly and hourly, respectively, data that were recorded at the nearest time point to the foragers’ outbound trip timestamp were used.

### 2.5 Meteorological variables

Daily meteorological data including temperature (°C), humidity (%), wind speed (km/hr), and cloud cover measured at the nearest time point to the foragers’ outbound trip in the Xiangshan park area, Beijing for the study period were retrieved online from CustomWeather, Inc. (timeanddate.com). Since bees can still navigate as long as there are clear patches of sky, cloud cover was categorized into 2 levels simply; “nonovercast,” and “overcast.”

### 2.6 Statistical analysis

All statistical analyses were performed using R version 3. 2. 5 (R Core Team, 2016). The Tukey Honest Significant Differences test (ANOVA Tukey multiple comparisons) was conducted using the R function *TukeyHSD* to compare group differences of mean foraging trip durations during the predust storm, dust storm, and postdust storm periods. Due to a maintenance issue, we were unable to collect data for May 2, so this date was omitted in the predust storm period. Average foraging trip duration for each period and *p*‐values of the ANOVA Tukey multiple comparisons were calculated through 10,000 parametric bootstrap replicates.

We determined the effect of the predictor variables (DR, fine PM mass concentration and meteorological variables) on foraging trip duration of honeybee using a generalized linear model (GLM). The GLM with Gamma family (link = log) was fitted using the R function *glm() in* package *lme4*. In order to eliminate possible impacts of the dust storm event on foragers’ fitness and to evaluate pure effects of air quality on foraging performance, data obtained during poststorm period were excluded in the model (*N* = 138). Predicted probabilities on foraging trip duration from the model against one of the independent variables (IVs) for a given value of other IVs were calculated using package *TeachingDemos*. Interaction effects of fine PM mass concentration and overcast sky on the foraging duration were plotted using package *interactions*. Different models were evaluated using Akaike's criteria (Table S1) using package *bbmle*. Multicollinearity between the predictor variables was assessed by the variance inflation factor (VIF) using the R function *vif()* in package *car* (Table S2).

## 3 RESULTS

**FIGURE 1:**
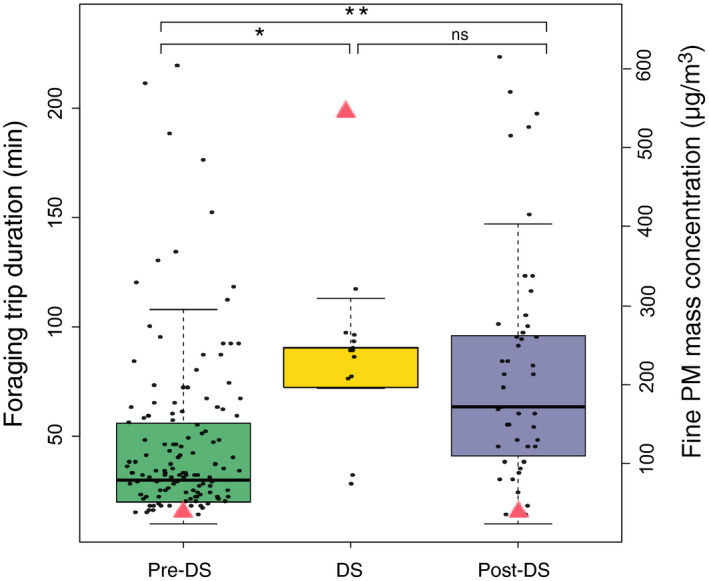
Foraging duration (min) of *Apis mellifera* foraging trips (*N* = 181) during predust storm (Pre‐DS, April 27–May 3), dust storm (DS, May 4), and postdust storm (Post‐DS, May 5–7) period. Daily foraging trip durations were resampled 10,000 times. Note that only a few observations (*n* = 12) were available during DS. 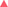 indicates the mean fine PM mass concentration of Pre‐DS (48 µg/m^3^), DS (573 µg/m^3^), and Post‐DS (49 µg/m^3^) period. Hourly fine PM mass concentrations between the earliest and latest foraging activity recorded of each day were averaged. * and ** denote significance as *p* < .05 and *p* < .001, respectively, by ANOVA Tukey multiple comparisons of means 95% family‐wise confidence level. ns: not significant (Table S3)

The foragers spent approximately 71% additional time than their previous average foraging duration on the heavily polluted day. Interestingly, the bees still invested 71% more time (on average) in foraging after the dust storm had swept through than before the dust storm (Figure 1). When the hourly fine PM mass concentration reached its maximum on May 4th, the daily average foraging duration was approximately 77 min. This was approximately 32 min longer than the daily average prior to the dust storm. It was clearly shown that the average foraging durations between the dust storm day and postdust storm days were not significantly different (Table S3). However, the average foraging durations both during the dust storm and after the dust storm were significantly greater than predust storm levels (*p* < .05 and *p* < .001, respectively).

We determined the effects of the DR and fine PM mass concentration for each foraging trip recorded from April 27 (predust storm period) to May 4 (dust storm period) using a GLM (Table 1). The real‐time DR and fine PM mass concentration along with meteorological variables including cloud cover were included in the model as predictor variables.

**TABLE 1:** Effects of predictor variables (optical property (DR) and fine PM mass concentration, and meteorological variables) on foraging trip duration of individual foragers

	Estimate	Std.Error	*t*‐value	*p* value
(Intercept)	3.111	0.973	3.196	.002*
DR	−4.457	2.165	−2.059	.042*
Fine PM mass concentration	0.004	0.001	4.112	<.001**
Cloud cover (nonovercast)	0.574	0.393	1.458	.147
Temperature	0.040	0.027	1.516	.132
Wind speed	0.007	0.034	0.213	.832
Humidity	−0.003	0.010	−0.281	.779
Fine PM mass concentration : Cloud cover	−0.027	0.013	−2.115	.036*

* and ** denote significance as *p* <.05 and *p* < .001, respectively.

**FIGURE 2:**
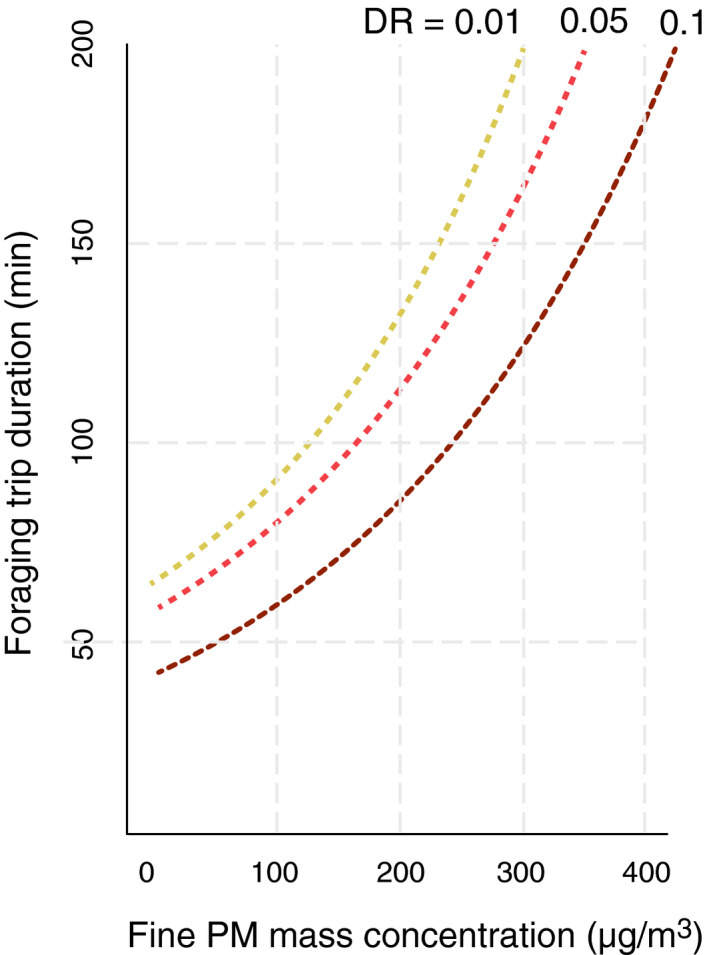
Predicted foraging trip duration (min) from the model against fine PM mass concentration for given values of the other predictors. Temperature = 28°C, Wind speed = 7 km/h, Humidity = 10%, Cloud‐cover = Overcast

**FIGURE 3:**
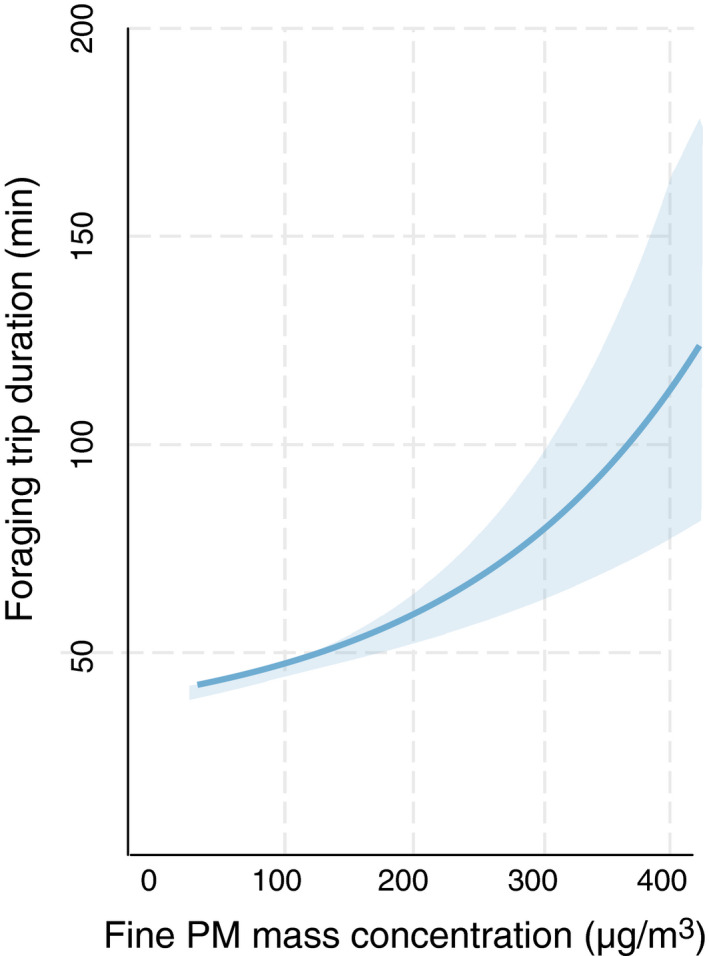
Predicted effects of fine PM mass concentration on foraging trip duration under overcast skies. All the other predictor variables are mean‐centered. The solid line indicates the mean slope estimate, and the shaded area is the predicted 95% confidence interval

In our results, the DR and fine PM mass concentrations were strongly associated with foraging duration (*p* = .042 and *p* < .001, respectively). As DR decreased, foraging duration became longer. This demonstrates that when the atmosphere was dominated by more spherical urban pollutants (smaller DR) than mineral dust (larger DR), the bees spent more time in foraging. Delays were predicted with a high mass concentration of fine PM. For given values of the other predictors, foraging trip duration increased with increasing fine PM mass concentration, irrespective of DR (Figure 2). Although cloud cover as a single predictor did not create a difference in honeybee foraging duration, a pronounced effect of fine PM mass concentration was found in the overcast sky (*p* = .036, Figure 3). Meteorological factors were not associated with time spent for foraging by bees.

## 4 DISCUSSION

Foraging trip duration was strongly associated with fine PM mass concentration, meaning that days with high fine PM mass concentrations can have a profound impact on honeybee foraging performance regardless of a big dust storm event. High fine PM mass concentrations typify a severe urban pollution day.

In a limited number of studies, the relation between the fine PM and DoP has been discussed. For example, a wildfires outbreak can reduce the DoP due to multiple scatterings of smoke aerosols (Hegedüs et al., 2007a; Shaw et al., 2014). According to a study by Hegedüs et al. (2007a), the average DoP was lower than 8% during a forest fire outbreak which is below the threshold necessary for navigation for bees.

Although the fine PM mass concentration alone can have a significant effect on foraging duration, this effect is synergistic with an overcast sky. Polarization pattern (the angle of polarization) is rather uniform under different cloud conditions (Hegedüs et al., 2007b; Pomozi et al., 2001). However, the DoP is reduced when the sky is overcast, which means that the extent of celestial polarization information to be useful for bees becomes very limited. Under completely overcast skies with thick clouds, the DoP can drop to zero (Brines & Gould, 1982; Pomozi et al., 2001) . Therefore, it is logical the effect of fine PM mass concentration is synergistic with overcast skies, which supports our results. This implies that honeybees will experience considerably more difficulties in navigating under very cloudy sky when air quality is poor.

Further, the result demonstrates that the smaller the DR is, the longer the expected foraging duration is, which is opposed to our hypothesis. In this study, foraging duration increased in an anthropogenic pollutant‐dominant case (smaller DR) rather than a dust‐dominant one (larger DR). The DR provides useful information to characterize the dominant particle type in the atmosphere in terms of their physical shape (Bi et al., 2017; Ge et al., 2011). During an Asian dust event, nonspherical particles are predominantly distributed in the atmosphere. These irregularly shaped mineral particles have a large DR.

Although each of the DR and size describes a different optical property of PM, larger particles tend to have a larger DR. For instance, in an empirical study conducted in Beijing, the average hourly and monthly DR measured for coarse particles with optical size (Dp) of 5 µm was higher than fine particles with Dp of 1 µm (Tian et al., 2018). The DoP depends greatly on the size of scattering particles (Schechner et al., 2003), and larger particles can be less effective in terms of depolarization. Light scattering for a given mass concentration of PM increases with decreasing particle size (Hinds, 1999). Thus, fine mode particles (and their microphysical properties) have strong effects on the DoP of the sky (Boesche et al., 2006). In this reason, our visibility is also governed by fine mode PM (ranges from 0.1 to 2 µm). Therefore, effects on foraging trip duration by each DR and fine PM mass concentration can counter to each other.

This is in line with a study that explores how different combinations of aerosol mode and AOD influence the DoP. Over land surface with a given AOD, the average DoP of fine mode is lower than that of coarse mode characterized as polluted dust (Chen et al., 2020). Taken overall with our results, what is important in determining the DoP of the sky is not the dominant morphology but the mass concentration of fine mode particles. This possibly explains why foraging duration did not increase with increasing DR in this study. However, when foraging trip duration was regressed on DR only (alongside meteorological variables) in our study, DR did not show any significant effect while the opposite was true for the fine PM mass concentration (Table S4). Therefore, mass concentration of fine PM overrides the effect of DR on foraging performance of honeybee.

Given that the average foraging duration of disoriented colonies was significantly longer than oriented colonies in an experimental study (I’Anson Price et al., 2019), the increased trip duration observed in this study during a heavy air pollution episode was possibly attributed to the complexity of visual cues as expected.

It is noteworthy that the foraging duration of forager bees after the dust storm did not return to their predust storm levels. Though the average fine PM mass concentration of the postdust storm period (49 µg/m^3^) was as low as that of the predust storm mass concentration (48 µg/m^3^), the bees still spent 32 min more in foraging. This may be traced to a low quality of food foraged and physical damage they incurred during the dust event. Since newly stored (1‐day‐old) fresh pollen is 3 times more likely to be consumed than older stored (10‐day‐old) pollen (Carroll et al., 2017), a state of malnutrition among the colony may persist if low‐quality food resources are stored and consumed for a few days. However, because we did not look into variations in quality or availability of floral resources through the study period, any causal link between foraging trip duration and resource availability depending different air quality should be considered in further studies.

In a recent study conducted in India where there are many of the world most polluted cities, significant correlations between PM10 increases and physiological changes of Giant Asian honeybees (*Apis dorsata*) were found (Thimmegowda et al., 2020). Giant Asian honeybee samples from severely polluted areas (in Bangalore, India) showed significantly lower rates of survival. Also, the bees from the highly polluted sites were more exposed to toxic metals such as lead (Pb). Serious physical damages on wings, antennae, and hindlegs were observed as well. More importantly, colony‐level chronic impacts through gene expression could also be predicted. For example, vitellogenin, associated with survival of worker bees, was found depleted in bees sampled from the highly polluted site compared with those from the low polluted site (Thimmegowda et al., 2020). This is important because the reduced fitness of individual foragers and their colony due to degraded foraging performance may have detrimental impacts on pollination services.

The experimental design of this study was restricted owing to an outbreak of intense air pollution during the study period. In addition, we note that our results may not reflect nor specify the presence of certain types of air pollutants such as nitrogen dioxide and hydroxyl radicals. For instance, Fuentes et al. (2016) studied the effects of air pollutants such as ozone on floral scents. They found that floral volatile compounds were greatly degraded even when the atmosphere was moderately polluted. Honeybee foragers took longer to locate floral sources even in very low O_3_ levels (less than 20 parts per billion on a per volume basis). However, in general, relatively short sunshine durations during a heavy pollution episode may prevent ozone precursors such as hydroxyl radicals from producing surface O_3_ (Lee et al., 2004). Interactions between different pollutant types can elicit different effects on honeybee foraging.

Though substantial attention has been paid to the pollination crisis in the US and Europe (Goulson et al., 2015; Teichroew et al., 2017), insufficient attention has been paid to the Asian countries that are also facing a crisis. In Asia, pollinator dependency is increasing in terms of crop yield (Potts et al., 2016) while air quality remains low persistently. For example, declines in some economically significant bee species have been locally reported in China (Teichroew et al., 2017). However, since air pollutants travel between not only countries but also continents, making a full circuit (Lee et al., 2019; Uno et al., 2009), the pollination crisis attributable to poor air quality should not be a localized issue. Continued climate change is also anticipated to increase airborne fine PM. Though effects of climate change on fine PM vary region to region, substantial increase in its mass concentrations are predicted in sources regions and more populated areas (Fang et al. 2013; Silva et al. 2017). In addition, under a warming climate, increases in frequency and severity of wildfires are expected to increase fine PM emissions in many regions (Schuur et al. 2015; Liu et al. 2016; Wotton et al. 2017).

Despite a lack of literature to correlate worsening air quality with bee population decline, the interplay of air pollution with other plausible stressors can amplify such a risk. For instance, the longer foragers search for food and navigate between their home and resources, the more likely they are to encounter other stressors such as insecticide residue and parasites (Fuentes et al., 2016).

Overall, our results lead us to conclude that the foraging performance of individual honeybees could be impeded due to low air quality. This is the first empirical study seeking to quantify variations in foraging duration that depend on air quality, while also analyzing foraging duration with respect to optical property of the atmosphere. Reduced foraging performance can be another stress adding to existing stressors that are believed to be the most proximate causes of the global bee decline.

## CONFLICT OF INTEREST

None declared.

## AUTHOR CONTRIBUTIONS

**Yoori Cho:** Conceptualization (equal); data curation (equal); formal analysis (equal); investigation (equal); methodology (equal); resources (equal); software (equal); validation (equal); visualization (equal); writing–original draft (equal); writing–review and editing (equal). **Sujong Jeong:** Conceptualization (lead); funding acquisition (equal); project administration (equal); supervision (equal); writing–original draft (equal); writing–review and editing (equal). **Dowon Lee:** Conceptualization (equal); supervision; writing–original draft (equal); writing–review and editing. **Sang‐Woo Kim:** Data curation (equal); methodology (equal); writing–original draft (equal). **Rokjin J. Park :** Investigation (equal); writing–original draft (equal); writing–review and editing. **Luke Gibson:** Investigation (equal); validation (equal); writing–original draft (equal). **Chunmiao Zheng:** Writing–original draft (equal); writing–review and editing. **Chan‐Ryul Park:** Supervision; writing–original draft (equal).

## ETHICAL APPROVAL

*Apis mellifera* used in this research is neither an endangered nor protected species.

## Supporting information

Table S1‐S4

## Data Availability

Data available from Figshare: https://doi.org/10.6084/m9.figshare.13347143.v1. Data including depolarization ratio and PM2.5 mass concentration are available from the authors upon reasonable request and with permission of the Asian dust and aerosol lidar observation network. Meteorological data is publicly available (CustomWeather, Inc. timeanddate.com).
