# Supplementary material for: Foraging trip duration of honeybee increases during a poor air quality episode and the increase persists thereafter
Source: Ecol Evol. 2021 Jan 23;11(4):1492–500. doi: 10.1002/ece3.7145 (PMC7882926; doi:10.1002/ece3.7145)
Supplement: Supplementary file 1 — Table S1‐S4 [file ECE3-11-1492-s001.docx]

Supplementary Information for

**Foraging trip duration of honeybee increases during a poor air quality episode and the increase persists thereafter**

**Yoori Cho, Sujong Jeong*, Dowon Lee, Sang-Woo Kim, Rokjin J Park, Luke Gibson, Chunmiao Zheng, and Chan-Ryul Park**

Correspondence to: [sujong@snu.ac.kr](mailto:sujong@snu.ac.kr)

**This PDF file includes:**

Supplementary Table 1

Supplementary Table 2

Supplementary Table 3

Supplementary Table 4

References

Supplementary Table 1 Different models were evaluated using Akaike’s criteria. The best-fitting model with lower Akaike information criterion (AIC) is in bold.

| Model(GLM, family=Gamma, Link=log) | $\Delta$AIC | df | $\Delta$AIC weight | Residual deviance |
| --- | --- | --- | --- | --- |
| **FD ~ DR + Fine PM * C + T + W + H** | **0.0** | **9** | **0.469** | **59.655** |
| FD ~ DR * Fine PM + C + T + W + H | 5.3 | 9 | 0.034 | 61.814 |
| FD ~ Fine PM + C + T + W + H | 6.0 | 7 | 0.023 | 63.828 |
| FD ~ DR * Fine PM * C + T + W + H | 3.5 | 12 | 0.081 | 58.661 |
| FD ~ DR + Fine PM + C + T + W + H | 3.3 | 8 | 0.092 | 61.815 |
| FD ~ DR + C + T + W + H | 15.6 | 7 | <0.021 | 68.073 |
| FD ~ Fine PM + DR * C + T + W + H | 1.6 | 9 | 0.210 | 60.307 |
| FD ~ Fine PM * C + T + W + H | 3.3 | 8 | 0.091 | 61.827 |

- Abbreviations: Fine PM, Fine PM mass concentration; DR, Depolarization ratio; C, Cloud-cover; T, Temperature; W, Wind speed; H, Humidity.

Supplementary Table 2 Detecting multicollinearity between predictor variables of different models based on Variance Inflation Factor (VIF). VIF smaller than 10 suggests the model does not have a collinearity issue^1^.

| Model | Predictor variable | | | | | | |
| --- | --- | --- | --- | --- | --- | --- | --- |
| FD ~ DR + Fine PM * C + T+ W + H | **Fine PM** | **DR** | **C** | **T** | **W** | **H** | **Fine PM:C** |
|  | **3.59** | **5.61** | **6.79** | **2.79** | **1.83** | **3.67** | **3.94** |
| FD ~ Fine PM + DR * C + T + W+ H | Fine PM | DR | C | T | W | H | DR:C |
|  | 3.69 | 6.21 | 39.46 | 2.43 | 1.73 | 3.86 | 35.42 |
| FD ~ DR * Fine PM + C + T + W + H | Fine PM | DR | C | T | W | H | Fine PM:DR |
|  | 117.48 | 6.66 | 3.10 | 2.61 | 1.53 | 5.60 | 137.71 |

Supplementary Table 3. Average foraging duration significantly increased on the day of DS outbreak and was not recovered to the pre-DS level even after the event ceased. (A) Average foraging duration (min) of individual foragers (*N=181*) between April 27 – May 7. (B) ANOVA Tukey multiple comparisons of means 95% family-wise confidence level (after Fligner-Killeen’s test). * and ** indicate *P* < 0.05 and *P* < 0.001, respectively. Daily foraging duration was resampled 10,000 times.

A

| Average Foraging Duration (min) | Pre-DS | DS | Post-DS |
| --- | --- | --- | --- |
|  | 45.04±11.30 | 76.74±7.18 | 76.55±15.39 |

B

| Group | Difference | Lower value | Upper value | *P value* |
| --- | --- | --- | --- | --- |
| Pre-DS/DS | -31.56 | -61.85 | -1.26 | **<0.05*** |
| Pre-DS/Post-DS | -31.20 | -48.47 | -13.93 | **<0.001**** |
| Post-DS/DS | -0.36 | -32.86 | 32.15 | **0.1** |

**Supplementary Table 4.** **Effects of each DR, Fine PM mass concentration, and cloud-cover on foraging duration alongside meteorological variables evaluated by GLMs (family = Gamma, log = link). * and ** indicate *P*<0.05 and *P*<0.001, respectively.**

1. Model: FD ~ DR + T + W + H ($\Delta$*AIC* : 13.9)

|  | Estimate | Std.Error | t-value | P value |
| --- | --- | --- | --- | --- |
| (Intercept) | 3.223 | 0.756 | 4.264 | **<0.001**** |
| DR | 1.085 | 1.117 | 0.972 | 0.333 |
| T | 0.002 | 0.023 | 0.087 | 0.931 |
| W | 0.064 | 0.031 | 2.087 | **0.039*** |
| H | 0.002 | 0.007 | 0.262 | 0.794 |

1. Model: FD ~ Fine PM + T + W + H *(*$\Delta$*AIC* : 6.1)

|  | Estimate | Std.Error | t-value | P value |
| --- | --- | --- | --- | --- |
| (Intercept) | 2.849 | 0.665 | 4.287 | **<0.001**** |
| Fine PM | 0.001 | 0.0005 | 2.502 | **0.014*** |
| T | 0.021 | 0.023 | 0.918 | 0.360 |
| W | 0.050 | 0.030 | 1.657 | 0.100 |
| H | 0.002 | 0.007 | 0.298 | 0.766 |

1. Model: FD ~ C + T + W + H ($\Delta$AIC : 15.1)

|  | Estimate | Std.Error | t-value | P value |
| --- | --- | --- | --- | --- |
| (Intercept) | 3.755 | 0.624 | 6.017 | **<0.001**** |
| C | -0.088 | 0.192 | -0.455 | 0.650 |
| T | -0.010 | 0.021 | -0.474 | 0.637 |
| W | 0.071 | 0.032 | 2.195 | **0.030*** |
| H | -0.002 | 0.007 | -0.292 | 0.770 |

References

1 Naimi, B. & Araújo, M. B. sdm: a reproducible and extensible R platform for species distribution modelling. *Ecography* **39**, 368-375, doi:10.1111/ecog.01881 (2016).
